# Risk Factors Associated with Complications and Early Mortality of Hip Fracture Surgery in Elderly Patients

**DOI:** 10.3390/medicina62050825

**Published:** 2026-04-27

**Authors:** Povilas Masionis, Giedrius Vaitukaitis, Agnietė Masionienė, Valentinas Uvarovas, Igoris Šatkauskas

**Affiliations:** 1 Faculty of Medicine, Institute of Clinical Medicine, Clinic of Rheumatology, Orthopaedic Traumatology and Reconstructive Surgery, Centre of Orthopedics and Traumatology, Vilnius Republican University Hospital, Vilnius University, LT-01513 Vilnius, Lithuania; giedrius.vaitukaitis@rvul.lt (G.V.); valentinas.uvarovas@rvul.lt (V.U.); igoris.satkauskas@rvul.lt (I.Š.); 2 Department of Anaesthesiology, Vilnius Republican University Hospital, LT-04129 Vilnius, Lithuania; agniete.masioniene@rvul.lt

**Keywords:** hip fracture, hemiarthroplasty, total hip arthroplasty, osteosynthesis, mortality

## Abstract

*Background and Objectives*: High rates of mortality and morbidity among elderly hip fracture patients are a recognized global issue. This study aimed to evaluate risk factors for early complications and 30-day mortality in hip fracture patients. *Materials and Methods*: The prospective study included 583 patients over 65 years old who sustained hip fractures from fall and underwent surgery. Each patient was followed up for 30 days and complications were recorded. Regression models were used to assess the influence of patient characteristics and laboratory markers on 30-day mortality and complications. *Results*: Any complication increased the risk of mortality by 5.6 times (95% CI 1.6–19.9, *p* = 0.008). Having > 6 comorbidities increased the risk of mortality by 8.2 (95% CI 1.9–35.5, *p* = 0.005) and the risk of complications by 2.3 (95% CI 1.9–35.5, *p* = 0.000). Patients > 85 years old had increased risk of mortality by 2.2 times (95% CI 1.2–4.1, *p* = 0.015) and a 1.7-fold increase in risk of complications (95% CI 1.2–2.4, *p* = 0.005). Vitamin D significantly predicted mortality with odds ratio of 2.1 (95% CI 1.1–4.1, *p* = 0.028). Serum N-terminal pro-brain natriuretic peptide levels > 780 ng/L predicted 2.3-fold increase in mortality (95% CI 1.0–4.9, *p* = 0.040) and a 2.6-fold risk of complications (95% CI 1.7–3.9, *p* = 0.000). *Conclusions*: Occurrence of complication increases the risk of mortality. Age and comorbidities are significant factors associated with 30-day mortality and complications. Vitamin D levels are associated with higher risk of mortality. N-terminal pro-brain natriuretic peptide levels correspond to higher risks of death and complications.

## 1. Introduction

Due to the ageing global population, it is estimated that 6 million people worldwide will experience hip fractures (HF) annually by the end of the century [1]. These patients often present with multiple comorbidities, such as hypertension, diabetes, obesity, heart failure, osteoporosis, alcohol addiction, and sarcopenia, and most cases require surgical intervention [1,2,3,4,5]. Early mortality rates among HF patients can reach up to 14%, and post-operative complication rates can reach up to 48% [6,7,8,9,10]. Given the fragility of these patients, identifying those at highest risk for adverse outcomes is important for determining the need for additional perioperative interventions [11]. Assessing factors associated with increased early mortality and morbidity may enhance post-operative outcomes through earlier identification and intervention for high-risk individuals [6,7,12,13,14,15]. This approach also clarifies surgical risks for both clinicians and patients and supports communication with patients’ relatives.

The aim of this study was to evaluate risk factors for early complications and 30-day mortality in elderly patients with hip fractures.

## 2. Materials and Methods

**Figure 1:**
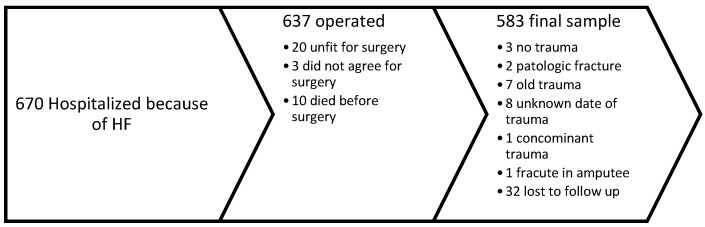
Flow chart of the final sample size.

A prospective study was carried out at a tertiary orthopedic trauma centre from 1 December 2022 to 31 December 2023. All participants or their proxies signed informed consent, and the study received approval from the Institutional Review Board and the regional bioethics committee. Inclusion criteria included patients aged 65 years or older; diagnosis of intertrochanteric, subtrochanteric, or femoral neck fracture; surgical treatment; low-energy trauma (simple fall); closed fractures; isolated trauma; and acute trauma (within 72 h). All surgeries were performed or supervised by a consultant orthopedic surgeon. Postoperative follow-up was conducted for 30 days. On day 30, investigators contacted the patient or proxy for an interview, and electronic medical records were reviewed. The following complications were documented: pneumonia, cardiac complications, surgical site infection, urinary tract infection, deep venous thrombosis, acute renal failure, readmission, reoperation, death, sepsis, delirium, and pressure sores. Additional data collected included age, gender, ASA classification, and use of direct oral anticoagulants (DOAC). Blood samples were obtained prior to surgery, as well as on the first and third or fourth postoperative days. Preoperative tests included hemoglobin (Hgb), C-reactive protein (CRP), troponin, N-terminal pro-brain natriuretic peptide (NT-proBNP), fibrinogen, total protein, albumin, and vitamin D. Hgb and CRP levels were measured again on the first and third or fourth postoperative days, and NT-proBNP was repeated on the third or fourth postoperative day. General patient data are summarized in Table 1. The final sample size of 583 patients is shown in Figure 1.

All analyses were performed using IBM SPSS version 26.0 software (SPSS Inc., Chicago, IL, USA) for Windows. Data that were normally distributed are reported as mean (±standard deviation). The Shapiro–Wilk test was utilized to assess the normality of data distribution. Multinomial regression models were constructed to evaluate the influence of patient characteristics (age, gender, fracture type, number of comorbidities, use of DOACs, and ASA class) and laboratory markers (preoperative Hgb, CRP, NT-proBNP, troponin, vitamin D, fibrinogen, albumin, and total protein concentrations; postoperative Hgb, CRP, and NT-proBNP concentrations on days 1 and 3–4) on early 30-day mortality and complications. An additional model was developed to assess the impact of each complication on early mortality. Individual models were generated for the evaluation of complications, patient factors and laboratory markers. Early death was set as a dependent variable in all models. Complications were set as dependent variables in models for patient factors and laboratory markers, but each specific complication was tested as an independent variable in the model for mortality prediction. Patient factors and laboratory markers were set as independent factors and converted to dummy variables. Specific ranges of dummy variables for continuous data are presented in the tables in the results section. The level of significance α was set at *p* < 0.05. Following initial modelling, deep venous thrombosis and fracture type were excluded from the models due to insufficient sample size—variables became redundant and had to be removed from analysis. Furthermore, the fracture type variable was eliminated because analysis without subtrochanteric fractures would be illogical.

## 3. Results

Incidence of complications is presented in Table 2.

Results regarding the complications’ influence on early mortality after HP surgery are presented in Table 3. The occurrence of any complication statistically significantly, by 5.6 times (95% CI 1.6–19.9), increased the risk of 30-day mortality of HF patients (*p* = 0.008). Of all the complications, only cardiac events showed a statistically significant (*p* = 0.010) odds ratio of 3.6 (95% CI 1.3–8.5).

Regression model results for patient data predicting early mortality and complications are presented in Table 4. The number of comorbidities and age showed a statistically significant effect on early mortality (*p* = 0.005 and *p* = 0.015 respectively). More than six diseases increased risk of 30-day mortality by 8.2 times (95% CI 1.9–35.5) and the risk of early complications was increased by 2.3 times (95% CI 1.5–3.7, *p* = 0.000). Patients older than 85 years had an increased risk of 30-day mortality by 2.2 times (95% CI 1.2–4.1) and the risk of early serious complications increased by 1.7 times (95% CI 1.2–2.4, *p* = 0.005).

Laboratory markers’ performance regarding early mortality and complications is presented in Table 5. Vitamin D concentration showed a statistically significant (*p* = 0.028) prediction, with a 2.1 odds ratio (95% CI 1.1–4.1), of early mortality. Furthermore, admission NT-proBNP showed a statistically significant prediction of early mortality and complication (*p* = 0.040 and *p* = 0.000 respectively). Before surgery, > 780 ng/L serum levels predicted a 2.3 times increase (95% CI 1.0–4.9) in 30-day mortality and 2.6 times increase (95% CI 1.7–3.9) in the risk of early complications. Baseline CRP > 10 mg/L showed a statistically significant (*p* = 0.011) predicted 1.7 increase in the risk of 30-day complications (95% CI 1.1–2.6).

## 4. Discussion

The data indicate that age and comorbidities are significant factors associated with 30-day mortality and complications in HP patients. The occurrence of any complication statistically significantly, by 5.6 times, increases the risk of early mortality and the risk of only cardiac complications also reached the level of significance. Patients aged over 85 present a 2.2-fold increased risk of early mortality and a 1.7-fold higher likelihood of postoperative complications. Having more than six comorbidities is linked to an 8.2-fold greater risk of early death and a 2.3-fold higher risk of complications. Regarding laboratory markers, vitamin D levels below 20 ng/mL are associated with a 2.1-fold increase in early mortality risk. Elevated baseline NT-proBNP corresponds with a 2.3-fold rise in early death risk and a 2.6-fold increase in the risk of complications. Higher baseline CRP levels are also associated with an increased likelihood of early complications. Age and comorbidities are established risk factors for early mortality in HF patients, highlighting the relevance of a quantitative evaluation in our HP cohort [1,6,16].

We applied a threshold of 781 ng/L for NT-proBNP based on the findings of Zhang et al., who followed 1354 patients and identified this value as predictive of all-cause mortality [1]. Although their mean follow-up was 34 months and the threshold was primarily associated with mid-term mortality, our study demonstrated applicability to early mortality within our cohort. Furthermore, there are limited studies evaluating the prognostic capabilities of NT-proBNP in HP patients. Despite methodological differences—some investigations identify threshold values while others categorize patients by quartiles or tertiles of NT-proBNP levels—the consensus is that pro-BNP concentrations exceeding 741–842 ng/L serve as valuable predictors of mortality in HP patients and should be routinely assessed [17,18].

Our study agrees with the results of previous research—low vitamin D levels in the blood have predictive value in showing early mortality in hip fracture patients [9,19,20]. The definition of vitamin D deficiency highly varies across the literature. In this study, the following classification ranges were used: (1) severe (<10 ng/mL), moderate (10–19.9 ng/mL), insufficient (20–29.9 ng/mL), and normal (≥30 ng/mL); (2) less than 50 nmol/L; (3) deficient <12 ng/mL, inadequate 12–<20 ng/mL; (4) insufficiency 20–<30 ng/mL and normal ≥30 ng/mL. We used < 20 ng/mL purely for methodological reasons. Furthermore, the exact mechanism of how vitamin D concentrations impact early mortality is not understood, but there are a few possible mechanisms. It was found that vitamin D has a negative correlation with albumin blood levels and they both might present with malnutrition and sarcopenia, which leads to adverse results; one the other hand, they are both negative acute phase reactants and may lead to dysregulation of the inflammation process [21,22,23]. Both mechanisms support the use of albumin levels as a confounder rather than evaluating vitamin D’s predictive value [19]. One of the most important factors is that vitamin D concentration is modifiable risk factor and requires intervention way before incidence of fracture.

Both preoperative and postoperative C-reactive protein levels have previously been identified as predictors of early and one-year mortality following HP surgery [24,25]. In our study, preoperative CRP was associated with early complications; however, we did not observe statistically significant results regarding its ability to predict early mortality. Notably, earlier studies on early mortality were retrospective in nature and utilized smaller sample sizes compared to the present analysis [24]. Additionally, our cohort included only patients with acute fractures (hospitalized within 72 h of trauma), which may have contributed to the lower observed CRP values. Methodological differences and varying cut-off points may also account for discrepancies between studies. As systemic inflammation is recognized as a predictor of hip fracture outcomes, our finding that CRP does not predict early mortality should be considered inconclusive.

Like other studies, this research has certain limitations. First, as a single-centre study conducted with one hospital cohort, the results may not be generalizable to the broader population. Second, fracture type and deep venous thrombosis could not be assessed in our models. In the case of subtrochanteric fractures, there were too few death events and too small a sample size for analysis; therefore, this variable became redundant. Subsequentially, all fracture types had to be removed because the analysis without one type is incorrect. We encountered the same problem with deep venous thrombosis—only seven cases were registered and the variable had to be removed from the model. These two maters potentially indicate that the sample size of 583 patients was not large enough. Furthermore, we had 32 cases of patients lost to follow-up, which might have an impact on the results. In all cases we could not contact the patient or his proxy and access the patient’s electronical health data—there is a good possibility that lost patients experienced complications or even death, but we were not able to confirm this. Furthermore, our multinomial regression model has a few potential flaws: (1) death is often the final outcome of complications, and it might be arguable that complications are an independent variable; (2) we did not match the recommended ratio of cases-to-variables, which indicates the low sample size. However, we found age and number of comorbidities to be significant predictors, which, in our opinion, shows that the models were capable.

## 5. Conclusions

The occurrence of any complication increased the risk of 30-day mortality in HF patient by 5.6 times. Only cardiac complications were significant predictors of early mortality. Patients aged over 85 present a 2.2-fold increased risk of early mortality and a 1.7-fold higher likelihood of postoperative complications. Having more than six comorbidities is linked to an 8.2-fold greater risk of early death and a 2.3-fold higher risk of complications. Vitamin D levels below 20 ng/mL are associated with a 2.1-fold increase in early mortality risk. Elevated baseline NT-proBNP corresponds with a 2.3-fold rise in early death risk and a 2.6-fold increase in the risk of complications. Higher baseline CRP levels are also associated with an increased likelihood of early complications.

## Figures and Tables

**Table 1:** General patient data.

Variables	
**Gender (female/male)**	452 (77.5%)/131 (22.5%)
**Age**	82 ± 8
**Number of comorbidities**	6 ± 3
**Fracture type:**	
Neck	292 (50.1%)
Pertrochanteric	259 (44.4%)
Subtrochanteric	32 (5.5%)
**Surgery type:**	
Hip hemiarthroplasty	191 (32.8)
Total hip arthroplasty	70 (12.0%)
Proximal femoral nail	236 (40.5%)
Dynamic hip screw	69 (11.8%)
Cannulated screws	17 (2.9%)
**ASA class:**	
ASA II	41 (7.0%)
ASA III	516 (88.5%)
ASA IV	26 (4.5%)
**DOAS use:**	124 (21.3%)

**Table 2:** Incidence of complications.

Variables	
All complications	207 (35.5%)
Pneumonia	66 (11.3%)
Cardiac complication	68 (11.7%)
Surgical site infection	20 (3.4%)
Urinary tract infection	58 (9.9%)
Deep venous thrombosis	7 (1.2%)
Acute renal failure	26 (4.5%)
Readmission	62 (10.6%)
Reoperation	11 (1.9%)
Death	47 (8.1%)
Discharge to nursery	269 (46.1%)
Sepsis	10 (1.7%)
Delirium	59 (10.1%)
Functional decline	406 (69.6%)
New mobility aid	556 (95.4%)
Pressure sores	51 (8.7%)

**Table 3:** Results regarding the complications’ influence on early mortality. * indicates significant result.

	Early Mortality	
Early Mortality	*p* Value	Odds Ratio (95% Confidence Interval)
Any complication	0.008 *	**5.6 (1.6–19.9)**
Pneumonia	*0.563*	1.3 (0.5–3.4)
Cardiac complication	0.010 *	**3.6 (1.3–8.5)**
Surgical site infection	*0.260*	0.2 (0.1–2.9)
Urinary infection	*0.535*	1.4 (0.5–3.9)
Renal failure	*0.504*	1.5 (0.4–5.1)
Readmission	*0.748*	0.8 (0.3–2.5)
Reoperation	*0.148*	5 (0.6–45)
Discharge to nursery	*0.561*	0.8 (0.4–1.8)
Sepsis	*0.899*	0.9 (0.1–6.1)
Delyrium	*0.130*	2.2 (0.8–6.1)
Pressure sores	*0.926*	1.1 (0.3–3.4)

**Table 4:** Regression model results for patient data predicting early mortality and complications. * indicates significant result.

	Early Mortality			Early Complications
	*p* Value	Odds Ratio (95% Confidence Interval)	*p* Value	Odds Ratio (95% Confidence Interval)
Gender	*0.371*	1.4 (0.7–2.9)	*0.692*	1.1 (0.7–1.7)
Comorbidities > 4	*0.277*	1.2 (0.6–2.9)	*0.752*	0.9 (0.3–2.1)
Comorbidities > 5	*0.209*	1.4 (0.7–3)	*0.395*	0.6 (0.2–2)
Comorbidities > 6	***0.005*** *	**8.2 (1.9–35.5)**	0.000 *	**2.3 (1.5–3.7)**
Age > 85	0.015 *	**2.2 (1.2–4.1)**	0.005 *	**1.7 (1.2–2.4)**
ASAII	*0.686*	1.7 (0.1–22)	*0.557*	0.7 (0.2–2.6)
ASAIII	*0.937*	1.1 (0.1–8.9)	*0.847*	1.1 (0.4–3.4)
ASAIV	*0.335*	3.1 (0.3–30)	*0.211*	2.4 (0.6–9.4)
DOAC	*0.798*	0.9 (0.4–1.9)	*0.667*	1.1 (0.7–1.7)

**Table 5:** Laboratory marker models’ results in the prediction of early mortality and complications. * indicates significant result.

	Early Mortality		Early Complications	
	*p* Value	Odds Ratio (95% Confidence Interval)	*p* Value	Odds Ratio (95% Confidence Interval)
HgB baseline < 100 g/L	*0.443*	0.7 (0.3–1.7)	*0.132*	0.6 (0.3–1.2)
HgB 1st day < 100 g/L	*0.476*	1.3 (0.6–3.1)	*0.435*	1.2 (0.8–1.9)
HgB 3–4th day < 100 g/L	*0.755*	1.1 (0.5–2.4)	*0.315*	0.8 (0.5–1.2)
Albumin < 35 g/L	*0.365*	0.7 (0.3–1.6)	*0.126*	1.6 (0.9–3)
Total protein < 60 g/L	*0.422*	0.7 (0.3–1.7)	*0.326*	0.7 (0.4–1.3)
Vitamin D < 20 ng/mL	0.028 *	**2.1 (1.1–4.1)**	*0.881*	1 (0.7–1.6)
NT-proBNP baseline > 780 ng/L	0.040 *	**2.3 (1–4.9)**	0.000 *	**2.6 (1.7–3.9)**
NT-proBNP 3–4th day > 780 ng/L	*0.308*	0.7 (0.3–1.4)	*0.531*	0.9 (0.6–1)
CRP baseline > 10 mg/L	*0.335*	1.4 (0.7–3)	0.011 *	**1.7 (1.1–2.7)**
CRP 1st day > 50 g/L	*0.334*	1.3 (0.7–2.7)	*0.244*	1.3 (0.9–1.8)
CRP 3–4th day > 50 g/L	*0.130*	1.7 (0.9–3.5)	*0.625*	1.1 (0.7–1.6)
Troponin > 9 ng/L	*0.252*	1.7 (0.7–4.4)	*0.726*	0.9 (0.6–1.4)
Fibrinogen > 4 g/L	*0.154*	0.6 (0.3–1.2)	*0.230*	1.3 (0.8–2)

## Data Availability

Data available on request due to ethical restrictions.
